# LONGITUDINAL TRAJECTORIES OF CAREGIVING BENEFITS AMONG FAMILY CAREGIVERS OF OLDER ADULTS

**DOI:** 10.1093/geroni/igae098.1150

**Published:** 2024-12-31

**Authors:** Yongjing Ping, Jeremy Lim-Soh, Truls Østbye, Rahul Malhotra

**Affiliations:** Duke-NUS Medical School, Singapore, Singapore; Duke-NUS Medical School, Singapore, Singapore; Duke University, Durham, North Carolina, United States; Duke-NUS Medical School, Singapore, Singapore

## Abstract

Although caregiving is often associated with burden, family caregivers can gain benefits – such as satisfaction, increased self-worth, and improved outlook towards life – from caregiving, which are linked with better care-recipient and caregiver outcomes. Acknowledging the dynamic nature of caregiving, many studies have investigated longitudinal patterns of caregiving burden, but studies on trajectories of caregiving benefits are limited. Furthermore, understanding the heterogeneity in trajectories of caregiving benefits will help identify risk factors linked with trajectories indicating low benefits or protective factors linked with high benefits over time. We conducted group-based trajectory modelling on longitudinal data – gathered at four time points, at six-to-twelve-month intervals – on caregiving benefits (short Positive Aspects of Caregiving scale) from 274 family caregivers of older adults in Singapore. We identified three relatively stable trajectory groups – “fulfilled” (14%, highest benefits), “satisfied” (64%, second highest benefits), and “dissatisfied” (22%, lowest benefits). Relative to caregivers in the “fulfilled” group, those cohabiting with their care-recipients were less likely to be in the “satisfied” group, those more prepared for caregiving or supported by experienced migrant domestic workers were less likely to be in the “dissatisfied” group, and those caring for care-recipients with more behavioral impairments were more likely to be in the “dissatisfied” group. Our findings provide evidence for heterogeneity in trajectories of caregiving benefits, indicate that many caregivers maintain relatively high caregiving benefits over time, and suggest that preparing migrant domestic workers and family caregivers for their caregiving role may contribute towards higher caregiving benefits over time.

